# High-Fat and Resveratrol Supplemented Diets Modulate Adenosine Receptors in the Cerebral Cortex of C57BL/6J and SAMP8 Mice

**DOI:** 10.3390/nu13093040

**Published:** 2021-08-30

**Authors:** Alejandro Sánchez-Melgar, Pedro José Izquierdo-Ramírez, Verónica Palomera-Ávalos, Mercè Pallàs, José Luis Albasanz, Mairena Martín

**Affiliations:** 1 Regional Center of Biomedical Research, Department of Inorganic, Organic and Biochemistry, Faculty of Chemical and Technological Sciences, School of Medicine of Ciudad Real, Universidad de Castilla-La Mancha, 13071 Ciudad Real, Spain; alejandro.sanchez@uclm.es (A.S.-M.); pedrojose.izquierdo@alu.uclm.es (P.J.I.-R.); Mairena.Martin@uclm.es (M.M.); 2 Department of Pharmacology and Therapeutic Chemistry, Faculty of Pharmacy and Food Sciences, Institute of Neuroscience, University of Barcelona, 08028 Barcelona, Spain; vpalomera@hotmail.com (V.P.-Á.); pallas@ub.edu (M.P.)

**Keywords:** adenosine receptors, high-fat diet, resveratrol, animal model, cerebral cortex

## Abstract

Neurodegenerative disorders are devastating diseases in which aging is a major risk factor. High-fat diet (HFD) seems to contribute to cognition deterioration, but the underlying mechanisms are poorly understood. Moreover, resveratrol (RSV) has been reported to counteract the loss of cognition associated with age. Our study aimed to investigate whether the adenosinergic system and plasma membrane cholesterol are modulated by HFD and RSV in the cerebral cortex of C57BL/6J and SAMP8 mice. Results show that HFD induced increased A_1_R and A_2A_R densities in C57BL/6J, whereas this remained unchanged in SAMP8. Higher activity of 5′-Nucleotidase was found as a common effect induced by HFD in both mice strains. Furthermore, the effect of HFD and RSV on A_2B_R density was different depending on the mouse strain. RSV did not clearly counteract the HFD-induced effects on the adenosinergic system. Besides, no changes in free-cholesterol levels were detected in the plasma membrane of cerebral cortex in both strains. Taken together, our data suggest a different modulation of adenosine receptors depending on the mouse strain, not related to changes in plasma membrane cholesterol content.

## 1. Introduction

Aging is inevitable, and most countries are facing increased populations of older people in recent decades. Aging is the major risk factor for neurodegenerative diseases such as Alzheimer’s or Parkinson’s disease [1]. Besides, there is a higher prevalence of high-fat diets in the Western-world population leading to a higher risk of obesity. It has been suggested that there is an association between cognitive impairment and obesity or overweight [2] which might increase the incidence of neurodegeneration in elderly people in the future. High-fat diets have been implicated in the development of many diseases in the brain, including memory impairments, depression, and other neurodegeneration disorders (reviewed in [3,4]). In fact, a great deal of evidence has demonstrated that overweight or obesity may subsequently result in a loss in cognitive abilities in murine [5,6], human adults [7,8,9], and even children [10,11]. indicating that high-fat diet (HFD) might be a consistent risk factor for neurodegenerative or dementia-like disorders. However, the underlying mechanisms of how HFD may affect cognition are poorly understood.

Adenosine is a ubiquitous molecule widely distributed in the central and peripheral nervous systems. In the brain, it is considered as an endogenous neuromodulator and neuroprotective molecule by controlling neurotransmitter release into the synaptic cleft [12]. All the different functions of adenosine are mainly orchestrated by specific adenosine receptors, which have been classified into A_1_, A_2A_, A_2B_, and A_3_ receptors. A_1_R and A_3_R are coupled to an inhibitory Gi-protein, whereas A_2A_R and A_2B_R mainly act through a stimulatory Gs-protein [13]. Both A_1_ and A_2A_ receptors are the most abundant adenosine receptors in the CNS and an alteration has been described in their corresponding density in several neurological and neuropsychiatric disorders, including Alzheimer’s disease (AD) [14], Parkinson’s disease [15] and Schizophrenia [16]. Among all four adenosine receptors, the A_2A_ receptor has gained attention since its involvement was reported in cognitive impairment [17] and neuroinflammation [18]. Besides, pharmacological inhibition of A_2A_R seems to be mainly neuroprotective [17,19,20,21,22]. The A_2B_ receptor has been less commonly studied due to its lower affinity to the endogenous ligand (i.e., adenosine) and lower expression as compared to A_1_R and A_2A_R [13]. However, recent studies point out a novel role of this receptor in metabolism as it has been described that A_2B_R signaling is crucial for energy expenditure in muscle cells [23], although there is less information available about the metabolic role of A_2B_R in the brain.

Cholesterol located in the plasma membrane can recognize and access the ortho-steric binding site of adenosine A_2A_ receptors [24]. However, the role of cholesterol in the regulation of neurotransmission is still poorly understood. It is widely accepted that HFD can elevate the content of total cholesterol in blood serum. The flux of macromolecules through the blood–brain barrier (BBB) is severely limited to protect the brain. Nevertheless, it is known that in the context of neurodegeneration the BBB becomes more permeable and may allow a less selective cross of substances [25,26]. In this scenario, little is known about whether the serum cholesterol may cross the BBB and disrupt the homeostasis of this lipid in the brain. Some authors have reported that increased levels of total cholesterol in blood serum could be associated with reduced cognitive functions [27,28], suggesting that cholesterol might be a key participant during the neurodegeneration process. In addition, homeostasis of brain cholesterol has been reported to be defective in several neurodegenerative diseases, including AD pathology [29,30,31], but its potential consequences for these disorders are yet to be elucidated.

Another molecule able to modulate adenosinergic signaling [32,33] is resveratrol (RSV), a plant-derived nutraceutical found in peanuts, berries, grapes, and red wines with proven beneficial properties for different pathologies, including metabolic [34,35] and neurodegenerative diseases [36,37]. RSV supplementation in the diet could represent a possible preventive and therapeutic strategy against neurodegeneration associated with metabolic alterations. Nevertheless, the precise mode of action of this phytochemical remains to be elucidated and more investigations are needed to shed light on how RSV improves cognition. Recently, we have reported that RSV interacts with adenosine receptors as a non-selective agonist [32], being able to modulate adenosine-mediated signaling in the brain upon long-term RSV supplementation in the diet [33]. This polyphenol did not change the body weight gain when compared to age-matched untreated SAMP8 mice [33]. HFD induces changes in body weight gain, glucose homeostasis in blood serum, and produces molecular changes in the hippocampus and cognitive decline in C57BL/6J [38], as well as in SAMP8 mice [39].

Therefore, the present work aimed to analyze whether HFD, RSV, or their combination, may affect adenosinergic signaling in the brain cortex of C57BL/6J (a wild-type model) and SAMP8 (an accelerated aging and AD-like pathology model).

## 2. Materials and Methods

### 2.1. Animals and Diets

Male C57BL/6J mice (22 months old, *n* = 27) were randomized in four experimental groups. The normal diet group (ND-old, *n* = 4) had *ad libitum* access to a standard chow diet (2018 Teklad Global 18% Protein, 6% Fat, Rodent Diet; Harlan Teklad, Madison, WI, USA) and tap water. The Resveratrol group (RSV, *n* = 5) had free access to standard chow diet enriched with trans-resveratrol (1 g/Kg, *w*/*w*, Mega Resveratrol, Candlewood Stars, Inc., Danbury, CT, USA). The high-fat diet group (HFD, *n* = 6) received a diet consisting of a AIN-93G diet modified to provide 60% of calories from fat (HFD: carbohydrate:protein:fat ratio of 16:23:61%). A group with high-fat diet containing 1 g/Kg *w*/*w* resveratrol (HFD + RSV, *n* = 5) was also used. Resveratrol chow (both ND and HFD) was formulated to provide daily doses of ~160 mg/Kg to the mice (human equivalent dose of 12,97 mg/Kg body weight [40]). In addition, a fifth group of young (8 weeks) mice receiving a standard chow diet were added (ND-young, *n* = 7) as a young cohort. After eight weeks of treatment, animals were euthanized under anesthesia [38].

Male SAMP8 mice (*n* = 20) were randomized into three groups at 6 weeks of age and following 15 weeks of treatment were euthanized under anesthesia. Normal diet (ND, *n* = 6) receiving a standard AIN-93G diet (carbohydrate:protein:fat ratio of 64:19:17%), high-fat diet (HFD, *n* = 7) and high-fat diet supplemented with RSV (HFD + RSV, *n* = 7) receiving the modified AIN-93G diet (carbohydrate:protein:fat ratio of 16:23:61%) [39].

The different diet duration for each strain was established based on the cognitive decline observed for each (supplemental Figure 1). After the sacrifice of animals, brain cortical regions were isolated and stored at −80 °C until experimentation was carried out. Mice were treated according to European Community Council Directive 86/609/EEC and were approved by the Institutional Animal Care and Use Committee of the University of Barcelona (670/14/8102, approved at 14 November 2014) and by Generalitat de Catalunya, Spain (10291, approved at 28 January 2018). Every effort was made to minimize animal suffering and to reduce the number of animals used in this study.

### 2.2. Plasma Membrane Isolation

Plasma membranes of the cerebral cortex of C57BL/6J and SAMP8 mice were isolated as previously described [33]. Samples were homogenized in isolation buffer (50 mM Tris-HCl, pH 7.4, containing 10 mM MgCl_2_ and protease inhibitors) in a Dounce homogenizer (10× pestle A, 10× pestle B). Samples were then centrifuged at 1000× *g* for 5 min in a Beckman JA 21 and the supernatant was centrifuged at 27,000× *g* for 30 min. The obtained pellet, considered as the plasma membrane fraction, was resuspended in the isolation buffer. Samples were stored at −80 °C until needed. The concentration of protein was quantified by the Lowry method using bovine serum albumin as standard.

### 2.3. 5′-Nucleotidase Activity Assay

5′-Nucleotidase activity (5’NT) was measured as previously described [41]. Briefly, 20 µg of protein from plasma membrane fraction were preincubated for 10 min at 37 °C in the reaction medium (50 mM Tris-HCl, 5 mM MgCl_2_, pH 9). Then, the reaction was started by adding AMP (final concentration of 500 µM). After 20 min, the reaction was stopped by adding 10% trichloroacetic acid (TCA). Samples were placed on ice for 10 min and centrifuged at 12,000× *g* at 4 °C for 4 min. The supernatants were used to measure inorganic phosphate released using KH_2_PO_4_ as Pi standard following the protocol described by Chan et al. [42]. The nonenzymatic hydrolysis of AMP was corrected by adding samples after TCA. Protein concentration and incubation times were selected to ensure the linearity of the reactions. Enzymatic activity was expressed as nanomolar Pi released/min mg protein. All samples were run in duplicate.

### 2.4. Adenosine Receptors Quantification by Western Blotting Assay

Plasma membrane proteins from each sample (20 µg) were mixed with loading buffer (0,125 M Tris, 20% glycerol, 10% mercapto-ethanol, 4% SDS, and 0.002% bromophenol blue, pH 6.8) and heated at 50 °C for 5 min. After electrophoresis on 10% SDS–PAGE gel in a mini-protean system (Bio-Rad, Madrid, Spain), samples were transferred to nitrocellulose membranes in the iBlot^TM^ Dry Blotting System (Invitrogen, Madrid, Spain). Membranes were then washed with PBS-Tween 20, blocked with PBS containing 5% skimmed milk, and incubated at 4 °C overnight with the primary antibodies at 1:1000 dilution for anti-A_1_R (Abcam, ab124780), 1:1000 for anti-A_2B_R (Merck-Millipore, ab1589p), and 1:2000 for anti-GAPDH used as a gel loading control (Abcam, ab8245). After rinsing, the membranes were incubated with the corresponding secondary antibody (GARPO 172-1019 or GAMPO 170-6516 from Bio-Rad) at a dilution of 1:4000 in PBS containing 5% skimmed milk for 1 h. Protein bands were detected using the ECL chemiluminescence detection kit (Amersham, Madrid, Spain) in a G:Box chamber, and specific bands were quantified with the GeneTools software (Syngene, Cambridge, UK). Molecular weight standards were from Bio-Rad.

### 2.5. Radioligand Binding Assays

Radioligand binding assays were performed in the plasma membrane fraction as previously described [33]. Plasma membranes were incubated with 5 U/mL Adenosine Deaminase in 50 mM Tris, 2 mM MgCl_2_, pH 7.4, at 37 °C for 30 min, to remove endogenous adenosine. Then, plasma membranes (50 µg) were incubated for 2 h at 25 °C with a saturated concentration of 20 nM [^3^H]ZM 241385. Theophylline (3 mM) was used as displacing ligand to obtain non-specific binding. The assay was stopped by rapid filtration through Whatman GF/B filters pre-incubated with 0.3% polyethyleneimine using a FilterMate Harvester (Perkin Elmer). Radioactivity was measured in a Microbeta Trilux (Perkin Elmer) liquid scintillation counter. Each sample was performed in duplicate.

### 2.6. Free Cholesterol Quantification

Free cholesterol (FC) was quantified following the manufacturer’s indications (MAK043, Sigma-Aldrich). 20 µL of plasma membrane fraction of each sample was added into 200 µL of a mixture containing Chloroform:Isopropanol:IGEPAL (7:11:0.1) for cholesterol extraction from samples. Then, samples were centrifuged at 13,000× *g* for 10 min and supernatants were transferred into new tubes. Supernatants were heated at 50 °C for 40 min to eliminate the organic phase from samples. Next, samples were resuspended in the corresponding assay buffer. After mixing 25 µL of resuspended samples with 25 µL of a reaction mix, 96-wells plate was incubated for 1 h at 37 °C and protected from light. Absorbance was then measured at 570 nm and interpolated into a standard curve. Data were then normalized to the amount of protein and represented as µg of free cholesterol/µg protein.

### 2.7. Statistical and Data Analysis

Data are represented as mean ± SEM in each graph. Differences between mean values were considered statistically significant at *p* < 0.05. One-way ANOVA was performed for statistical analysis, as indicated in the figure captions, with the GraphPad Prism 7.0 program (GraphPad Software, San Diego, CA, USA).

## 3. Results

### 3.1. 5′-Nucleotidase Activity in the Cerebral Cortex

**Figure 1:**
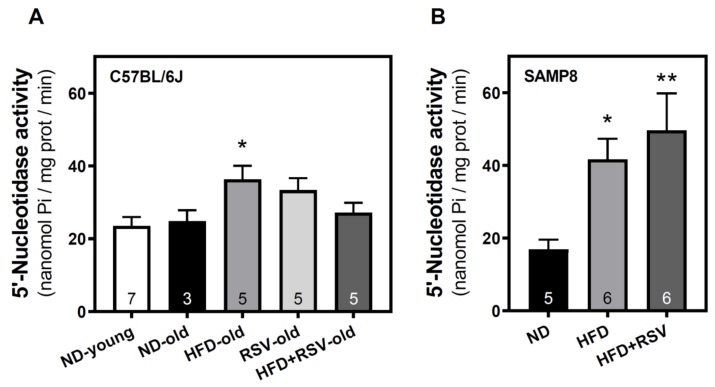
5′-Nucleotidase activity in the cerebral cortex. The plasma membrane fraction was used in order to assess the activity of 5′-nucleotidase in the cerebral cortex of C57BL/6J (**A**) and SAMP8 (**B**) mice. Enzymatic activity was measured as described in Methods. Data are the mean ± SEM of three-seven different samples (indicated within the bars). * *p* < 0.05 and ** *p* < 0.01 significantly different from ND-old experimental group in panel **A** or from ND cohort in Panel **B** according to one-way ANOVA.

To evaluate whether adenosinergic signaling is modulated by HFD we first analyzed the adenosine-generating enzyme 5′nucleotidase activity located at the plasma membrane. HFD increased the activity of this enzyme in C57BL/6J (Figure 1A) and SAMP8 (Figure 1B) mice when compared to their corresponding age-matched ND group. In turn, no differences were found in this enzymatic activity in C57BL/6J (Figure 1A) and SAMP8 mice (Figure 1B), when comparing HFD and HFD + RSV groups.

### 3.2. Adenosine A_1_, A_2A_ and A_2B_ Receptors Level in the Cerebral Cortex

**Figure 2:**
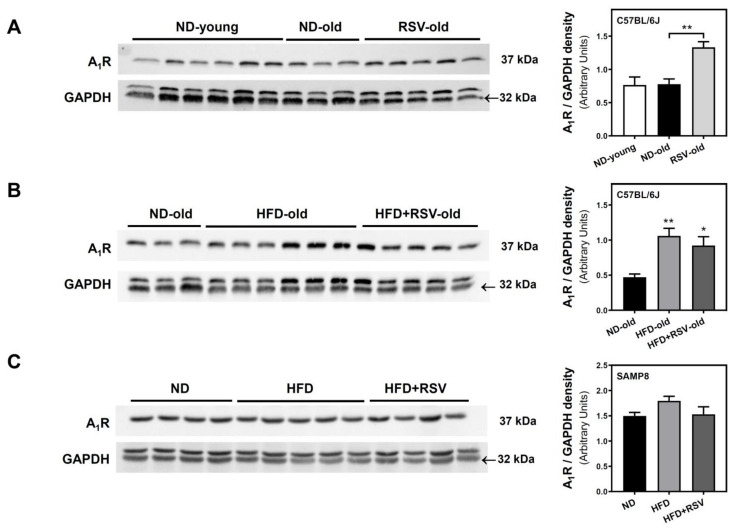
Adenosine A_1_ receptor levels in the cerebral cortex. A_1_Rs were quantified by Western blotting assay in plasma membrane fraction carried out as described in Methods. Figures represent the level of this receptor in the cerebral cortex of C57BL/6J (**A**,**B**) and SAMP8 (**C**) mice. Results are mean ± SEM of three-six different samples. GAPDH was used as a loading control. The upper GAPDH band correspond to the A_1_R band. * *p* < 0.05 and ** *p* < 0.01 significantly different from ND-old group according to one-way ANOVA.

Once we detected an alteration of 5′-nucleotidase activity induced by HFD in both mice strains, we carried out Western blotting and radioligand binding assays to verify whether HFD was also able to affect adenosine receptors density located at the plasma membrane fraction. In C57BL/6J mice, the density of A_1_R was similar in both ND-young and ND-old groups (Figure 2A). However, RSV, HFD, and HFD + RSV diets caused a significant increase in A_1_R level when compared to the ND-old group (Figure 2A,B). On the contrary, no significant changes were observed in A_1_R levels either in HFD or HFD + RSV groups in SAMP8 mice as compared to the ND group (Figure 2C). In addition, RSV (HFD + RSV group) did not change the HFD diet effect in any mice strain (Figure 2B,C).

**Figure 3:**
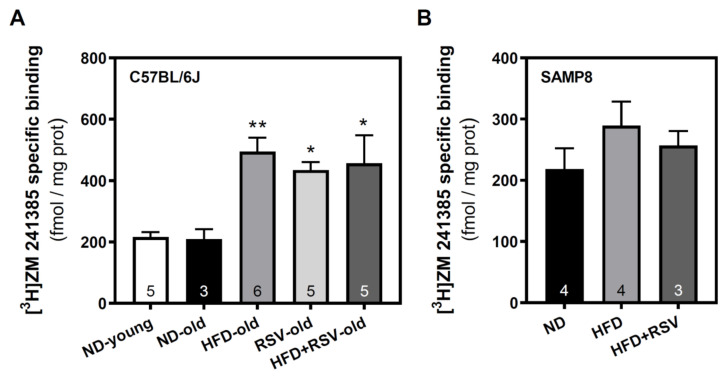
Adenosine A_2A_ receptor level in the cerebral cortex. Radioligand binding assays were carried out in plasma membrane fractions of C57BL/6J (**A**) and SAMP8 (**B**) mice to assess the modulation of this receptor. [^3^H]ZM 241385, at a saturated concentration of 20 nM, was used as selective radioligand and nonspecific binding was determined in the presence of 3 mM theophylline as described in Methods. Data are mean ± SEM of three-six different samples (indicated within the bars). * *p* < 0.05 and ** *p* < 0.01 significantly different from ND-old group according to one-way ANOVA.

The possible modulation of A_2A_R levels was analyzed by radioligand binding assay. In C57BL/6J, all diets (RSV, HFD, and HFD + RSV) caused a significant increase in A_2A_R specific binding when compared to the ND-old group. However, RSV (HFD + RSV group) did not change the HFD diet effect (Figure 3A). In contrast, in SAMP8 mice HFD alone or combined with RSV did not induce any significant change in A_2A_R levels as compared to the ND group (Figure 3B).

**Figure 4:**
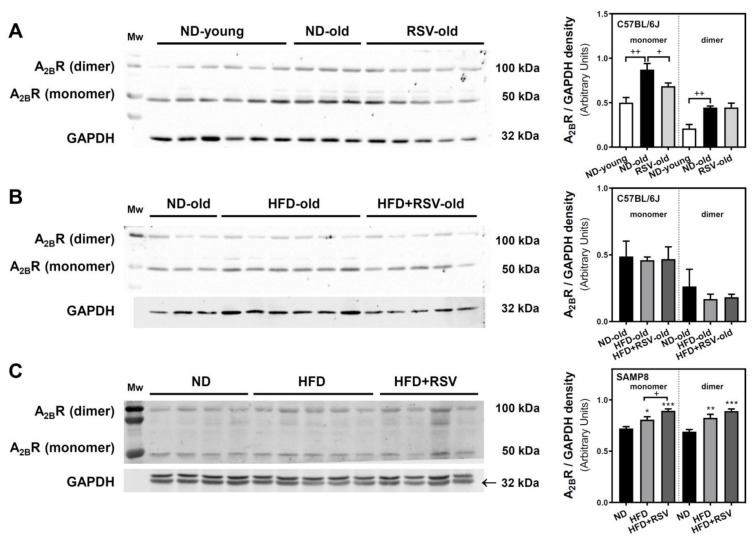
Adenosine A_2B_ receptor density in the cerebral cortex. Monomeric and dimeric forms of A_2B_R were detected by Western blotting assay in the plasma membrane fraction of cerebral cortex of C57BL/6J (**A**,**B**) and SAMP8 (**C**) mice. Data are mean ± SEM of three-six different samples. GAPDH was used as a gel loading control. The upper GAPDH band in panel **C** correspond to the A_1_R band. + *p* < 0.05 and ++ *p* < 0.01 significantly different from indicated bars according to one-way ANOVA. * *p* < 0.05, ** *p* < 0.01 and *** *p* < 0.001 significantly different from ND group (panel **C**) according to one-way ANOVA.

We next analyzed the A_2B_R by Western blotting, which revealed two bands: a lower band at 50 kDa that might represent the receptor in its monomeric form, and a less intense band detected at 100 kDa which could represent the detection of A_2B_R homodimer. In C57BL/6J mice, both monomeric and dimeric forms of A_2B_R were higher in the ND-old group than in the ND-young group, suggesting an age-related increase in the expression of this receptor. However, a significant reduction in the monomeric form of A_2B_R was found in the RSV group. This reduction was not detected in the dimeric form (Figure 4A).

No significant changes were detected in C57BL/6J mice receiving HFD or HFD + RSV diets (Figure 4B). However, in SAMP8 mice, the monomeric and dimeric forms of A_2B_R were increased in HFD and HFD + RSV as compared to ND experimental group (Figure 4C). In addition, while RSV (HFD + RSV group) did not change the HFD diet effect in C57BL/6J mice (Figure 4B) it caused a significant increase of the monomeric form in SAMP8 mice (Figure 4C).

### 3.3. Adenylyl Cyclase Activity in the Cerebral Cortex

**Figure 5:**
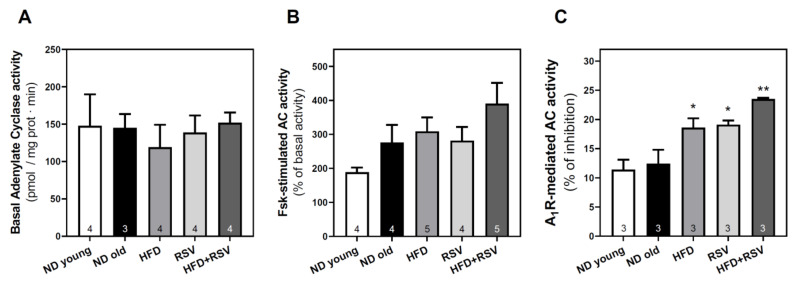
Adenylyl cyclase activity in the cerebral cortex of C57BL/6J mice. Basal (**A**), forskolin (Fsk)-stimulated (**B**) and A_1_R-mediated (**C**) adenylyl cyclase activities were measured in the plasma membrane fraction of the cerebral cortex of C57BL/6J mice. Data are mean ± SEM of three-five different samples (indicated within the bars). * *p* < 0.05 and ** *p* < 0.01 significantly different from ND-old group (panel **C**) according to one-way ANOVA.

The modulation of A_1_R, A_2A_R, and A_2B_R levels detected in C57BL/6J could result in the modulation of adenylyl cyclase (AC) activity, the main effector system of adenosine receptors. Therefore, this activity was only measured in the different experimental groups in C57BL/6J mice. Basal AC activity (Figure 5A) was similar in all experimental groups. Forskolin-stimulated AC activity (Figure 5B) did not change by diet as compared to the ND-old group. However, the inhibition of AC activity with CPA, a selective A_1_R agonist, was significantly increased by HFD, RSV, and HFD + RSV diets as compared to the ND-old group (Figure 5C), in agreement with the higher A_1_R levels found in these experimental groups. In addition, basal, forskolin-stimulated, or A_1_R-mediated AC activities in the HFD + RSV group were not significantly different from the corresponding values in the HFD group (Figure 5).

### 3.4. Level of Free Cholesterol in Plasma Membrane of Cortex Brain

**Figure 6:**
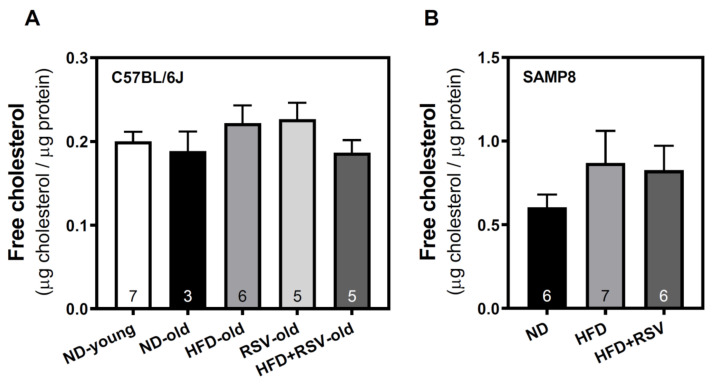
Level of non-esterified cholesterol in the plasma membrane of the cerebral cortex. The plasma membrane fraction was used to quantify cholesterol in the cerebral cortex from C57BL/6J (**A**) and SAMP8 (**B**) mice. Free cholesterol was measured as described in Methods. Data are the mean ± SEM of three-seven different samples (indicated within the bars).

Once we detected the modulation of different components of the adenosinergic system, mainly induced by HFD, we analyzed the cholesterol level in the plasma membrane in the different experimental groups. The presence of cholesterol in the plasma membrane was not significantly affected in the cerebral cortex of both C57BL/6J (Figure 6A) and SAMP8 (Figure 6B) mice, suggesting that changes in the level of adenosine receptors are not related to cholesterol.

## 4. Discussion

Wild-type C57BL/6J strain is frequently used as a mouse model of aging and neurodegenerative diseases [43,44,45], whereas SAMP8 strain could be an ideal candidate as a model of AD-like pathology due to the phenotypic hallmarks described in this animal model such as cognitive impairment, astrogliosis, Aβ accumulation, neuroinflammation, and oxidative stress, among others [46,47]. As early as 3 months of age, SAMP8 mice have impairments in spatial learning, aberrant gene expression, oxidative stress, and Tau hyperphosphorylation; at 5 months, impairment in spatial memory and increased tau hyperphosphorylation; at 6 months, hippocampal cognitive impairment, glial degeneration, inflammation, and Aβ deposition; and at 8 months, they present gliosis and increased levels of soluble Aβ [46,47,48]. The lifespan of SAMP8 mice is about 10–12 months [49,50] while C57BL/6J strain is about 26–28 months [51,52]. It has been previously reported that HFD induces cognitive impairment and molecular changes in the hippocampus of the same animals analyzed here as compared to their corresponding age-matched ND-cohort [38,39]. The hippocampus is related to memory formation and other cognitive functions, and it has been extensively studied in neurodegeneration, as reviewed elsewhere [53]. However, the cerebral cortex is also implicated in cognition and multiple molecular changes, including the modulation of adenosinergic signaling [14,54], have been reported in this brain area in AD [55,56].

Results presented herein (summarized in Table 1) indicate that adenosine receptors in the cerebral cortex undergo a different modulation by diet depending on the mouse strain. Thus, HFD or RSV induced the overexpression of A_1_R and A_2A_R in C57BL/6J mice, whereas no changes were observed in the SAMP8 strain. In contrast, A_2B_R seems to be more affected in SAMP8 than in C57BL/6J mice. Interestingly, higher activity of 5′-NT was found as a common effect induced by HFD in both experimental models. Besides, the combination of HFD plus RSV did not clearly modify the HFD-induced effect on adenosinergic components.

A_1_R and A_2A_R are predominantly located in the synapses and are implicated in the fine-tune regulation of neurotransmitters release [12]. These two receptors have been found to be differently altered during aging, but also in several brain regions of AD patients. Thus, an increased amount and activity of A_1_R and A_2A_R were detected in the frontal cortex [14,57]. On the contrary, a decreased expression and amount of A_1_R was found in the dentate gyrus (DG) and CA1 region from the hippocampus, while these receptors remained unchanged in the CA3 region [58]. Besides, a decreased amount of A_1_R was reported in the striatum [59] and in temporal and medial temporal cortices and thalamus [60] in AD patients. Concerning A_2A_R, an increased expression of these receptors has been found during aging [12] and in AD patients, where there is a higher density of A_2A_R in the hippocampus [61], frontal cortex [14], frontal white matter, frontal gray matter, and hippocampus/entorhinal cortex [62].

The upregulation of A_1_R in C57BL/6J mice fed with HFD, RSV, or their combination (HFD + RSV), could represent a compensatory mechanism against the upregulation of A_2A_R to restore the inhibitory tone. The A_1_R has been associated with a neuroprotective role due to its inhibitory functionality [12]. It has been suggested that overexpression and overactivity of A_2A_R may accelerate neurodegeneration [63]. Moreover, A_2A_R deserves special attention in numerous neurological disorders since its pharmacological inhibition prevents memory deficits [17,20,64,65,66]. Intake of HFD is associated with cognitive dysfunction, as recently reviewed in rodents [67], and recapitulates some AD-like features in mice [68]. In the present study, we found increased A_2A_R levels in HFD fed C57BL/6J mice, a diet that also induces hippocampal-dependent memory deficits in C57BL/6J mice treated since their weaning until 6 months of age [69] and favors the formation of βA depositions in the DG area of the hippocampus when treated since their weaning until 16 months of age [70]. Therefore, A_2A_R might be involved in the HFD-induced cognitive deficits in this mouse strain [38,39]. Additionally, the previously described colocation and physical association between 5′-ecto-Nucleotidase (5′-NT) and A_2A_R [71] deserves also our attention. Our data indicated a similar effect on 5′-NT activity and A_2A_R levels in those mice who received HFD, which might lead to a potentiated functionality of the 5′-NT-A_2A_R axis in C57BL/6J mice. Accordingly, 5′-NT activity in SAMP8 mice was also found to increase in HFD-treated animals. Despite the fact that A_2A_R levels were not significantly increased in this animal model, the increased activity of 5′-NT could suggest an overactivation of A_2A_R by the adenosine generated by 5′-NT, as previously reported [72]. All these data point out a potential role of 5′-NT-A_2A_R signaling in the HFD-induced cognitive deterioration. Neuroinflammation is a key factor that may contribute to the progression of neurodegenerative disorders [73,74,75]. Microglial 5′-NT-A_2A_R-mediated signaling modulates microglial immune-response in the brain [72,76], hence, this functional axis may represent a potential target to control neuroinflammation. HFD caused an increment of proinflammatory cytokines in the hippocampus of the animals analyzed here [38,39] and other animal models of AD-like pathology fed with HFD also developed neuroinflammation [77,78,79]. It has been reported that 5′-NT inactivation attenuated pro-inflammatory responses in microglia, including a reduction of proinflammatory cytokines [72].

While A_2A_R did not change significantly in SAMP8 mice, the A_2B_R level was significantly increased in both HFD and HFD + RSV groups. Intriguingly, the compensatory mechanism of elevating A_1_R did not occur as A_1_R levels remain unchanged in these experimental groups. We recently reported a significant age-related loss of A_1_R density and functionality in the whole brain from this mouse strain [33] which could explain the absence of such a compensatory mechanism that takes place in C57BL/6J but not in SAMP8 mice.

Our data revealed an increase of the monomeric and dimeric forms of A_2B_R in old versus young mice in the wild-type strain C57BL/6J, while these levels remained unchanged in the different old experimental groups. By contrast, HFD and HFD + RSV treatments caused a higher density of monomeric and dimeric forms of A_2B_R in SAMP8 mice, suggesting that HFD only modulates A_2B_R in this mouse strain. Increased density of A_2B_R associated with aging has been observed in the whole brain from SAMP8 mice [33]. Metabolic and oxidative stress seem to be distinctive markers for neurodegeneration and AD pathology and may contribute to its progression. A_2B_R signaling and associated heterodimerization in muscle cells are essential in energy expenditure under physiological conditions and its genetic depletion is involved in the observed metabolic decline associated with age [23]. In line with this, A_2B_R plays an important role in modulating glucose homeostasis and fat mass, and it has been proposed as a significant regulator of HFD-induced hallmarks of type 2 diabetes [80]. Moreover, gene deletion of A_2B_R has been suggested as a suitable model for metabolic syndrome [81]. However, the role of A_2B_R in metabolic stress occurring in the brain needs further research.

Inducing metabolic stress by HFD in aged C57BL/6J (24 months) led to cognitive disturbances as compared with age-matched controls and with young mice [38]. HFD also induced metabolic stress in SAMP8 with impairment in cognitive capabilities, oxidative stress increase, and mitochondrial dysfunction [39]. In both murine models, the hippocampus was clearly affected by this metabolic stress which could also be extended to other brain regions such as the cerebral cortex, where we found an increased A_2B_R density in HFD and HFD + RSV groups. It has been reported that pharmacological inhibition of A_2B_R prevented neuronal death and synaptic failure upon oxygen and glucose deprivation in rat hippocampal slices independently of glutamate release [82]. In the context of neurodegeneration, injections of amyloid-β in Swiss male mice reduced the expression of A_2B_R and mitochondrial-related pathways, and the activation of A_2B_R with the agonist 5′-N-Ethylcarboxamidoadenosine (NECA) improved the mitochondrial function and integrity in different brain regions such as the hippocampus, pre-frontal cortex, and amygdala [83].

Based on RSV detection in the brain after intraperitoneal or oral administration, it has been reported that RSV crosses the BBB and reaches the brain of rodents [84,85] and humans [86]. The ability of resveratrol (RSV) to mitigate cognitive decline has been demonstrated by numerous studies in vivo [87,88,89,90]. RSV supplementation in the diet was able to counteract the HFD-induced cognitive failure in the same animals used in the present work [38,39]. RSV modulates the adenosinergic system in the whole brain of SAMP8 mice by potentiating A_1_R and desensitizing A_2A_R downstream pathways [33]. These effects can be due, at least in part, to a direct action of RSV as a non-selective agonist for adenosine receptors [32]. However, we did not observe a clear effect of RSV when mice were fed with HFD + RSV as compared to those SAMP8 mice who received HFD alone. A significant effect of RSV was only observed on A_2B_R levels which were increased even more in HFD + RSV than in the HFD group. Concerning C57BL/6J mice, although RSV alone can modulate some adenosinergic components (i.e., A_1_R, A_2A_R, A_2B_R, A_1_R-mediated AC inhibition), this polyphenol was unable to modify the effect of HFD. Therefore, the modulation of the adenosinergic system by RSV in the cerebral cortex seems not to be involved in the protective effect of RSV against the HFD-induced cognitive decline previously reported in these mice strains [38,39]. Further studies in other brain areas should be carried out to decipher the potential implication of adenosinergic signaling in the neuroprotective action of RSV.

Brain cholesterol metabolism seems to be defective in neurodegenerative diseases [91]. The cholesterol-24S-hydroxylase, a metabolizing enzyme responsible for the removal of cholesterol in neurons, and its product, 24-hydroxycholesterol, are reduced in the hippocampus of Alzheimer-like Tau pathology in mice [92], which could promote cholesterol accumulation in the CNS. However, disruption of the cholesterol 24-hydroxylase gene does not alter steady-state levels of cholesterol in the mouse brain [93]. It is well established that HFD can elevate total cholesterol in serum, which may constitute a risk factor for AD and contribute to the pathogenesis of this disorder [94,95]. It has been reported that brain cholesterol levels increase in mice fed with a cholesterol-rich diet [95], suggesting that BBB might be compromised and a less selective cross of substances may occur [91]. However, whether plasma cholesterol can cross the BBB under some circumstances, such as neurodegeneration, remains to be elucidated. Apart from changing membrane fluidity, cholesterol by enriching lipid rafts serves as a platform for signaling transduction of proteins in the plasma membrane and can allosterically modulate (positively or negatively) ligand binding and/or functional properties of different GPCRs [96]. Interestingly, plasma membrane cholesterol binds and accesses A_2A_R, and can modulate the A_2A_R levels in the cell surface of in vitro glial cells [24]. Therefore, changes in cholesterol levels might also cause the modulation of adenosine receptors in vivo. However, cholesterol levels are similar in all experimental groups assayed in C57BL/6J and SAMP8 mice, suggesting that the HFD-induced modulation of the adenosinergic system in C57BL/6J mice is cholesterol-independent, at least in the cerebral cortex.

## 5. Conclusions

Our study indicates that both HFD and RSV diets modulate adenosinergic signaling without altering free cholesterol levels in the plasma membrane of the brain cortex from C57BL/6J and SAMP8 mice. This modulation is dependent on the mouse strain, affecting A_1_R and A_2A_R in C57BL/6J mice and A_2B_R in SAMP8 mice. Besides, higher activity of 5′-NT was found as a common molecular effect induced by HFD in both animal models, suggesting that such an enzyme could be involved in the cognitive decline previously reported in the same animals.

## Figures and Tables

**Table 1:** Summary of changes detected in the cerebral cortex of C57BL/6J and SAMP8 mice.

	C57BL/6J					SAMP8		
Parameter	ND-Young	ND-Old	HFD-Old	RSV-Old	HFD + RSV-Old	ND	HFD	HFD + RSV
A_1_R	99	100	225 **	171 **	196 *	100	120	102
A_2A_R	103	100	236 **	208 *	218 *	100	132	117
A_2B_R-monomer	57 **	100	94	79 *	96	100	112 *	124 ***
A_2B_R-dimer	47 **	100	64	100	69	100	119 **	129 ***
5′-NT	95	100	146 *	134	109	100	246 *	293 **
Basal AC	102	100	82	96	105	--	--	--
A_1_R-mediated AC inhibition	91	100	150 *	154 *	190 **	--	--	--
cholesterol	106	100	118	120	99	100	143	136

Changes on the indicated parameters were detected in the different experimental groups of C57BL/6J and SAMP8 mice. Control groups (100%) were ND-old and ND for C57BL/6J and SAMP8, respectively. Data are the means represented as a percentage. * *p* < 0.05, ** *p* < 0.01, *** *p* < 0.001 significantly different from their corresponding control. Statistical differences were applied according to one-way ANOVA. --, not done.

## Data Availability

Data is contained within the article.
